# Experimental and Numerical Development on Multi-Material Joining Technology for Sandwich-Structured Composite Materials

**DOI:** 10.3390/ma14206005

**Published:** 2021-10-12

**Authors:** Lucian Zweifel, Igor Zhilyaev, Christian Brauner, Martin Rheme, Gregor Eckhard, Valentin Bersier, Slobodan Glavaški, Ricardo Pfeiffer

**Affiliations:** 1 Institute of Polymer Engineering, FHNW University of Applied Sciences and Arts Northwestern Switzerland, Klosterzelgstrasse 2, 5210 Windisch, Switzerland; igor.zhilyaev@fhnw.ch (I.Z.); christian.brauner@fhnw.ch (C.B.); 2 MultiMaterial-Welding AG, Zentralstrasse 115, 2503 Biel, Switzerland; martin.rheme@mm-welding.com (M.R.); gregor.eckhard@mm-welding.com (G.E.); valentin.bersier@mm-welding.com (V.B.); slobodan.glavaski@mm-welding.com (S.G.); 3 KVT-Fastening AG (Bossard Group), Lagerstrasse 8, 8953 Dietikon, Switzerland; R.Pfeiffer@kvt-fastening.com

**Keywords:** joining, process modelling and simulation, sandwich structures, numerical analysis, ultrasonic technology, polymer composites

## Abstract

Creating connection points for sandwich-structured composites without losing technical performance is key to realising optimal lightweight structures. The patented LiteWWeight^®^ technology presents cost-effective connections on sandwich panels in a fraction of a few seconds without predrilling. Ultrasonic equipment is used to insert a thermoplastic fastener into the substrate material and partially melt it into the porous internal structure. This creates a highly interlocked connection (connection strength is above 500 N) suitable for semi-structural applications. This study focused on the simulation and experimental validation of this process, mainly on the interaction between the pin and the substrate material during the joining process. The dynamic thermo-mechanical model showed reasonable agreement with experimental methods such as process data, high-speed camera monitoring or computed tomography and allowed the prediction of the connection quality by evaluation of the degree of interlock. The connection strength prediction by the developed model was validated within several various process setups, resulting in a prediction accuracy between 94–99% depending on the setup.

## 1. Introduction

The introduction of fibre-reinforced polymers in load-bearing automotive structures provides a great potential to significantly reduce weight, fuel consumption, and consequently CO_2_ emissions. Reliable and cost-effective joining technologies must be developed to reduce production time and enable the manufacture and assembly of composite structural parts. Here, sandwich-structured composite materials provide clear advantages in terms of weight savings and mechanical performance in the aerospace and automotive industries owing to their high stiffness and strength to weight ratios. Such structures consist of two thin high density face sheets, bonded to a thick core made from low density foam material. Variation of the materials combination and foam to void ratio allows production of lightweight structural systems with desirable resulting material properties.

Using appropriate materials for appropriate scenarios is becoming more popular in transportation areas such as in the automotive and railway sectors, leading to multi-material design concepts with a high demand for reliable, cost-efficient joining methods [1,2]. Nevertheless, traditional fastening methods (metallic fasteners, adhesive bonding) for joining fibre-reinforced polymers decrease mechanical performance [3,4]. Metallic fasteners have a relatively high weight. Additionally, their thermal expansion coefficients and thermal conductivities are higher than those of the surrounding composite structure, which could lead to composite degradation in the joint region with changes in the surrounding temperature. Furthermore, some metallic fasteners require holes to be drilled in advance, which could increase the chance of crack initiation in the joint area. Damage in the connection area produced by the conventional joining methods and further intensive loading conditions might debone the skin from the core and initiate the crack propagation [5,6].

Adhesive bonding is an alternative to metallic fasteners. However, these techniques strongly depend on the contacting surfaces morphology and presence of contaminants. Bonding processes are time-consuming and difficult to analyse once cured. Moreover, debonding leads to significant damage to the contact region [7,8]. Fibre-reinforced thermoplastic rivets have recently been implemented for joining composite structures [9,10]. During the joining process, thermoplastic rivets are heated above the melting temperature, which allows the creation of a squeeze flow of the rivet material into the internal composite structure. However, thermoplastic welding methods, such as ultrasonic welding, offer several advantages over the above-mentioned joining techniques, such as short process times and strong dependable joints without significant surface preparation efforts [8,11,12,13,14,15]. Ultrasonic welding is one of the most commonly used welding methods besides resistance welding [11,13,14] or conduction welding [16] for joining thermoplastic structures. It uses ultrasonic energy at high frequencies (20–40 kHz) to produce low peak-to-peak amplitude (50 to 100 μm) mechanical vibrations. In ultrasonic welding, the heat required to melt the thermoplastics is generated locally. This occurs through friction between the joining constituents and viscoelastic damping [17,18]. Ongoing research has focused on developing ultrasonic welding solutions to replace gluing and riveting for primary load-carrying aerospace structures [13,18,19]. These applications are generally larger structures based on reinforced high-performance thermoplastic materials, such as polyetheretherketone (PEEK), polyphenylene sulphide (PPS), and polyetherimide (PEI) [13,20,21]. Furthermore, continuous ultrasonic welding systems that are capable of continuously joining large flat and double-curved structures have been shown to be a fast and feasible welding technique [20,22].

**Figure 1:**
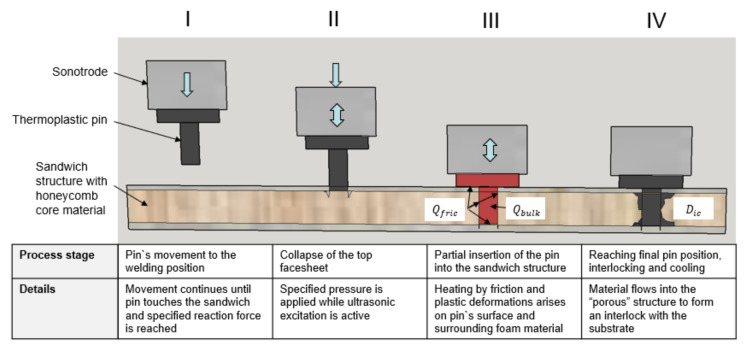
Schematic overview of the MM-Welding^®^ LiteWWeight^®^ process. Adapted from Ref. [15].

The MM-Welding^®^ LiteWWeight^®^ technology is especially designed for sandwich structures with honeycomb and foamed core materials (Figure 1). Ultrasonic energy is used to insert a thermoplastic connection element (pin) through the top layer into the porous structure of the core. Owing to the ultrasonic excitation, the thermoplastic connection element melts at the interface, infiltrates the porous structure, and creates a highly interlocked bond with the substrate material. The goal of this study is to adapt the knowledge-based approach for modelling the LiteWWeight^®^ process targeting interior automotive applications e.g., fixation elements on casing panels in the trunk.

The MM-Welding^®^ LiteWWeight^®^ process is divided into four phases:

- Phase 1: Ultrasonic device moves to target position to start joining process.
- Phase 2: Pressure is increased on the thermoplastic pin until the trigger force is reached that starts the ultrasonic excitation. The pressure continues to increase until the pin dynamically pierces the upper face sheet resulting in a local face sheet collapse.
- Phase 3: The thermoplastic pin moves through the substrate. Tolunay et al. [23] suggested that heating is solely due to mechanical dissipation of work through viscoelastic deformation. In the case of a flat energy director, the heating mechanism, as suggested by Zhang et al. [24] and Villegas et al. [17,18] also consists of frictional dissipation at the interface. The heat sources are based on two effects: bulk phenomena and friction-based interfacial heat sources. Based on available literature [23,25], heat generation appears in the material itself from the viscoelastic dissipation (bulk phenomena). This is based on the total or partial contact of the sonotrode (hammering effect), the loss modulus of the material, and the frequency and amplitude of the imposed strain.
- Phase 4: Intimate contact: During the insertion, the contact between the parent materials and connection element evolves as the surface of the connection element melts and flows/squeezed into the porous material. To take into account this effect Levy et al. [25] proposed an adapted model from Lee et al. [26] dependent on a dimensionless scalar definition that allows an assessment of the process quality.

This study aims to assess whether it is feasible to create a dynamic numerical impact model of the LiteWWeight^®^ process that allows an evaluation of the connection quality. The impact model calculates the stresses, strains, and damage rates for pin and sandwich structures. Additionally, the numerical model enables the calculation of heat from friction and plastic deformation. The model was verified and validated based on a comparison with experimental tests. The data provided by the ultrasonic device, such as force, displacement, and ultrasonic amplitude, depending on time, were used for the reconstruction of the sonotrode displacement boundary condition (BC). Computed tomography (CT) scans were used to reconstruct the sandwich geometry and pin insertion position. Furthermore, a high-speed camera was used to visually assess the LiteWWeight^®^ process and calculate the hammering coefficient. Additional studies were performed to evaluate the damage rate and the temperature distribution of the pin during joining.

## 2. Materials and Methods

### 2.1. Materials

**Thermoplastic material**: The thermoplastic pins were made from the fibre-reinforced polymer PA66 GF30 (BASF, Ultramid A3WG6, Ludwigshafen, Germany) and were produced by injection moulding. The use of a glass fibre-reinforced polymer is advantageous because of the increased Young’s modulus and mechanical loss factor of the material. Therefore, the ultrasonic vibrations were converted into heat more efficiently, which was crucial for the MM-Welding^®^ LiteWWeight^®^ process. The thermoplastic pins were dried at 90 °C for 8 h because of the influence of the absorbed humidity in PA66 GF30 [15]. The pins resulting elastic modulus is 5500 MPa at room temperature. Thus, a temperature-dependent elastic modulus was implemented into the numerical model.

**Sandwich-structured composite:** A lightweight panel, which is typically integrated in the automotive sector as an interior component, was used. It is based on face sheets made of glass fibre-reinforced polymers and a honeycomb core structure made of cardboard. The components were bonded together using a polyurethane resin that partially expanded into the core layer. The elastic modulus for the face sheet is 4550 MPa, 400 MPa for the honeycomb cardboard internal structure and 300 MPa for the foam. The overall thickness of the panel was 16 mm, whereby top and bottom face sheets were 0.5 mm. The length and width of a honeycomb element was respectively 13 mm and 6 mm.

### 2.2. Material Characterisation

Various methods have been used to characterise the mechanical and thermal material properties of the pin material PA66 GF30 and sandwich material to identify parameters for the finite-element simulation model. First, differential scanning calorimetry experiments were performed to analyse the crystallisation behaviour at different cooling rates and to derive heat capacities. Temperature- and frequency-dependent dynamic mechanical analysis measurements were performed for the purpose of storage and loss modulus. Furthermore, the following mechanical tests were conducted: tensile test on the face sheet substrate material, compression behaviour of the pins, and the sandwich substrate. The face sheet tensile test is essential for the implementation of the Hashin damage criteria. A detailed representation of the data is documented in Brauner et al. [15] and summarised in Appendix A (Table A1).

### 2.3. Ultrasonic Equipment and Process Monitoring Tools

In this study, a servo-driven ultrasonic welder (Dukane iQ Servo, Prague, Czech Republic) was used. The equipment monitored the force, displacement, amplitude, frequency, velocity, and energy with a sampling rate of 1000 Hz. The ultrasonic insertion of LiteWWeight^®^ pins was optimised in a preliminary study by systematic variation of relevant processing parameters using a design of experiments [15]. Hence, optimised reference process parameters were used in this study. A velocity of 45 mm s^−1^ and an amplitude of 76 μm operating at 20 kHz were applied.

**Figure 2:**
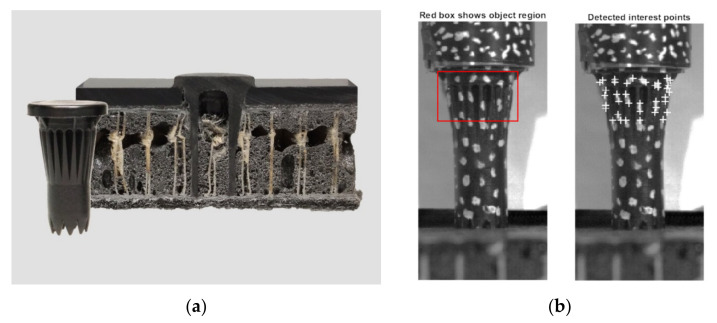
(**a**) Thermoplastic pin inserted in lightweight panel for high-strength fixation; (**b**) Detected points of interested on thermoplastic pin by usage of the Kanade–Lucas–Tomasi point tracking algorithm.

Because the LiteWWeight^®^ process is highly dynamic, high time resolution methods were exploited to allow further data analysis and interpretation. A FASTCAM SA5 high-speed camera (Photron, Tokyo, Japan) was used to analyse effects such as pin movement, hammering (contact between the pin and sonotrode), and potential pin-tip failure. To derive the concept of the hammering coefficient, which plays an important role in calculating heat by friction and dissipation, a MATLAB algorithm was developed and coupled with a high-speed camera to track various points on the pin and sonotrode through the process (Figure 2). High-speed videos were acquired at 75 kHz, which was the limit due to the imaging resolution and the available light sources. An ideal sinus wave at 20 kHz was considered during this process. Therefore, 3.75 points per cycle were obtained by high-speed measurements to characterise the sinusoidal wave. The Kanade–Lucas–Tomasi (KLT) algorithm implemented in MATLAB (Version 9.8, V Natick, MA, USA) Computer Vision Toolbox was used to track various points of the sonotrode and pin [27]. The algorithm returned the trajectories of positions that were successfully tracked throughout the process. The most suitable trajectory for both the sonotrode and pin was extracted depending on the quality and stability of the signal.

**Figure 3:**
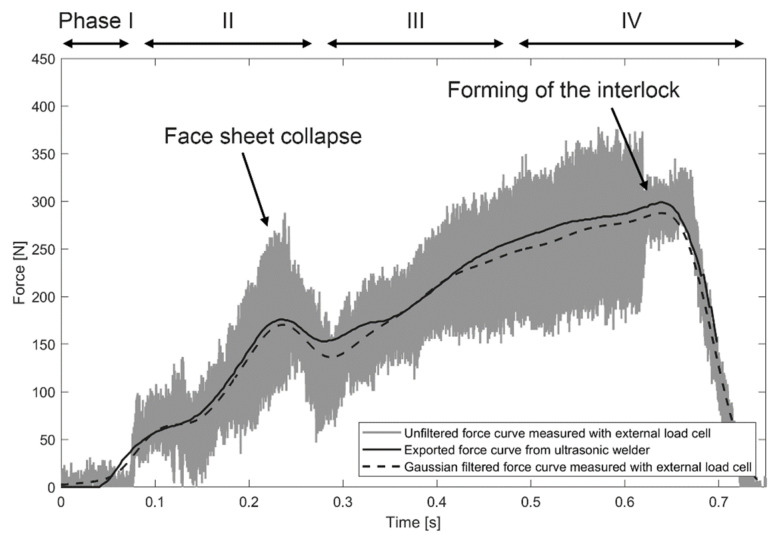
Comparison of force curves derived from different sensor data.

Additionally, the load cell KM40d (ME-Meßsysteme GmbH, Hennigsdorf, Germany) was used to record the reaction force below the sandwich panel with a high time resolution of 1 kHz. The unfiltered force curve results from oscillatory reaction forces combined with measurement errors due to high sampling rate. The measured force signal was smoothed using a Gaussian filter method (see Figure 3), which showed reasonable agreement with the exported machine data. Equivalent post-processing was applied to the reaction forces derived by the numerical model.

Additionally, CT scans were performed. The scans presented an inside view of the inserted pin, highlighted the mechanical strength of the pin and the surrounding foam content. Furthermore, it allowed quick measurement of the densities of the components used and showed possible weaknesses that were introduced during manufacturing (e.g., porosities within the pin due to injection moulding). The mechanical strength of the pin was measured by a pulling out procedure in a Zwick 100 kN universal tensile test machine (Zwick, Ulm, Germany) at a testing speed of 5 mm/min to determine the maximum force. Furthermore, a partial insertion study was performed with variations in insertion collapse length. This method allowed the assessment of the damage rate of the thermoplastic pin tips at various phases during the process.

### 2.4. Numerical Methods

The commercial software Abaqus (Version 6.13, Providence, RI, USA) was used for the simulation of the heat transfer, elastic and plastic deformations, and failure in pin and substrate elements. A fully explicit coupled thermal-mechanical dynamic analysis was implemented. This full coupling was needed because most of the heat arose from friction and dissipation, and the material properties are temperature-dependent.

#### 2.4.1. Thermo-Mechanical Model

Three general heat sources arose in the pin and substrate during the process: heat from friction, plastic deformation, and ultrasonic energy dissipation. The heat fluxes generated on the contacting surfaces of the pin and substrate were estimated according to the following:

(1) $$q_{p}=q_{k}-f_{1}q_{g},$$

(2) $$q_{s}=-q_{k}-f_{2}q_{g}.$$

Here, $q_{p}$ and $q_{s}$ are the heat fluxes generated on the pin and substrate contacting surfaces, respectively, $q_{k}$ is the heat flux due to conduction, and $q_{g}$ is the heat flux from frictional energy dissipation. $f_{1}=f_{2}=0.5$ are the fractions of evenly distributed heat generated on the first and second surfaces, respectively [28]. The values for $f_{1}$ and $f_{2}$ were set as equal values, because both joining partners are polymer materials and therefore, the thermal properties like heat capacity, density, and thermal conductivity are similar. The heat generated by friction in Abaqus/Explicit was calculated using the factor $\eta_{fr}$, which defines the fraction of frictional work converted to heat. The heat flux generated by the frictional heat generation is given by

(3) $$q_{g}=\eta_{fr}\tau \Delta s/\Delta t,$$

where τ  is the frictional stress, Δs is the incremental slip, and Δt is the incremental time. The heat flux due to conduction is assumed to be of the form

(4) $$q_{k}=\kappa \Delta \theta ,$$

where κ is the heat-transfer coefficient, and Δθ is the temperature difference between the two sides. In [28] it was shown that heat from energy dissipation appeared in the domain as a thermal source according to the following equation:

(5) $${\dot{Q}}_{bulk}=\frac{\alpha^{2}\omega E_{loss}\varepsilon :\varepsilon }{2},$$

where ${\dot{Q}}_{bulk}$ is the heat rate, α is the hammering coefficient, ω is the frequency, $E_{loss}$ is the elastic loss modulus, and ε is the strain tensor. According to the modelling and experimental results presented in [18,23], the heat from friction on the interface of joined parts was significantly higher than heat from energy dissipation. Therefore, heat from ultrasonic energy dissipation was neglected in the Abaqus model but was calculated in the next step using the evaluated values of hammering coefficient and strains depending on time.

Abaqus allows the calculation of the inelastic heat fraction, which defines the amount of heat generated by mechanical dissipation associated with plastic strain. This term can be introduced as a source of coupling for the thermal-mechanical analysis. The inelastic heat fraction plays a significant role in LiteWWeight^®^ technology, because extensive inelastic deformations occur rapidly in a material whose mechanical properties are temperature-dependent, and heat has no significant time to dissipate. The heat flux per unit volume was estimated according to the equation

(6) $$r^{pl}=\eta_{pl}\sigma :{\dot{\varepsilon }}^{pl}.$$

Here, $\eta_{pl}$ is a user-defined parameter, σ is the stress, and ${\dot{\varepsilon }}^{pl}$ is the rate of plastic straining. General contact was defined in Abaqus/Explicit for the simulation of contact and interaction problems. All surfaces were automatically defined. This default surface contained all exterior element faces, all analytical rigid surfaces, and all edges in the model, as well as the nodes attached to these faces and edges. The general contact algorithm activated and deactivated the contact faces and contact edges in the contact domain based on the failure status of the underlying elements.

The Coulomb friction model was implemented to carry shear stresses by the definition of critical shear stress, at which sliding of the surfaces started as a fraction $\mu_{fr}$ of the contact pressure.

(7) $$\tau_{crit}=\mu_{fr}p,$$

where $\mu_{fr}$ is the same in all directions. Contact pressure-overclosure relationships are defined as a linear function of the clearance between the surfaces. In a linear pressure-overclosure relationship, the surfaces transmit contact pressure when the overclosure between them, measured in the contact (normal) direction, is greater than zero.

Abaqus/Explicit predicts material progressive damage and failure based on the undamaged elastic-plastic response of the material, damage initiation criterion, and damage. Plastic stress-strain ratio is defined from the experiments for the pin, and plastic properties of honeycomb cells and foam are known as a range of possible values (plastic and damage properties of internal substrate structure is a subject of model calibration procedure). In both cases, the von Mises yield surface was used to define isotropic yielding. It is defined as the value of the uniaxial yield stress as a function of the uniaxial equivalent plastic strain.

The pin destroyed the upper face sheet and the internal structure of the sandwich panel through the substrate. Therefore, failure models and element removal criteria were defined and integrated into the model. Further governing equations related to Abaqus/Explicit were complemented as supplemental material.

#### 2.4.2. Assessment of the Connection Quality

The main goal of the LiteWWeight^®^ technology is to form an interlock between the pin and substrate and provide a high value of pullout force. The form-locking process is a function of the process parameters (pressure, temperature, and time) and the geometry of the contacting surfaces. A geometric description of the surfaces is critical for modelling. Surface topography can be described as a nonstationary random process [29] or fractal distribution [30]. With simplified approaches, the surface profile can be interpreted as a series of periodic sine or cosine functions with different amplitudes and frequencies. A model of intimate contact by Dara and Loos [31] represents the surface as a set of rectangles of different sizes. Lee and Springer [26] and Mantell and Springer [32] proposed models with simplified geometry represented as a series of rectangles of the same size. In this study, a simplified ‘squeeze flow’ model by Mantell and Springer [32] was implemented as

(8) $${\dot{D}}_{ic}={\left(\frac{D_{ic,0}}{D_{ic}}\right)}^{4}{\left(\frac{a_{0}}{b_{0}}\right)}^{2}\frac{p}{\mu \left(T\left(t\right)\right)}.$$

Here, $D_{ic}$ is the degree of interlock, $D_{ic,0}$ is its initial value, $\mu \left(T\left(t\right)\right)$ is the viscosity of the pin material in a liquid state, p is the pressure, and $a_{0}/b_{0}$ is the ratio derived from the surface roughness.

The calculation of the degree of interlock according to expression (8) for the LiteWWeight^®^ technology is not a trivial task because the interface surface is non-constant, elements of pin and substrate were deleted according to the defined element removal criteria, and the whole pin moves relative to the substrate. For the validation of the proposed approach for the performance quality prediction, five different insertion setups were considered: reference, faster insertion speed, lower insertion speed, higher US amplitude, and lower US amplitude. Three trials were performed per setup. The specifications are presented in Table 1.

An Anton-Paar MCR 300 rheometer was used to perform rheological measurements that were used to describe the temperature-dependent viscosity of PA66 GF30. A plate-plate configuration was used for the measurements. The plate diameter was 25 mm, with a gap thickness of approximately 1 mm. The samples were dried before testing for 12 h at 90 °C. Additionally, the samples were exposed to nitrogen during the measurement because of the accelerated thermal degradation of PA66 GF30 at high temperatures. Furthermore, the confocal laser microscope (Keyence VK-X1100) enabled the characterisation of the surface roughness at different locations on the pin surface. The surface roughness values were evaluated with one-dimensional line roughness measurements according to standard DIN EN ISO 4287.

## 3. Results and Experimental Validation

**Figure 4:**
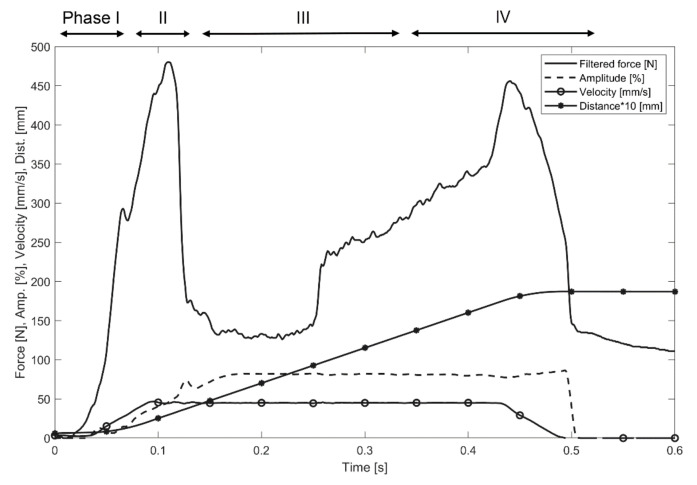
Process signals such as filtered force, amplitude, and velocity during the LiteWWeight^®^ joining process of a thermoplastic pin with the sandwich-structured composite panel.

The overall objective of this section is the validation of the numerical model by comparison with experimental results. The numerical model should adequately predict process performance which was represented by the degree of interlock that is dependent on the input parameters temperature, stress, and failure fields. After the insertion process, the most important process signals were exported from the ultrasonic welder, as shown in Figure 4. The force curve clearly presents phase 2 (first force peak, collapse of face sheet) and phase 4 (second force peak, forming the interlock) as described in Figure 1.

### 3.1. Geometry Reconstruction

**Figure 5:**
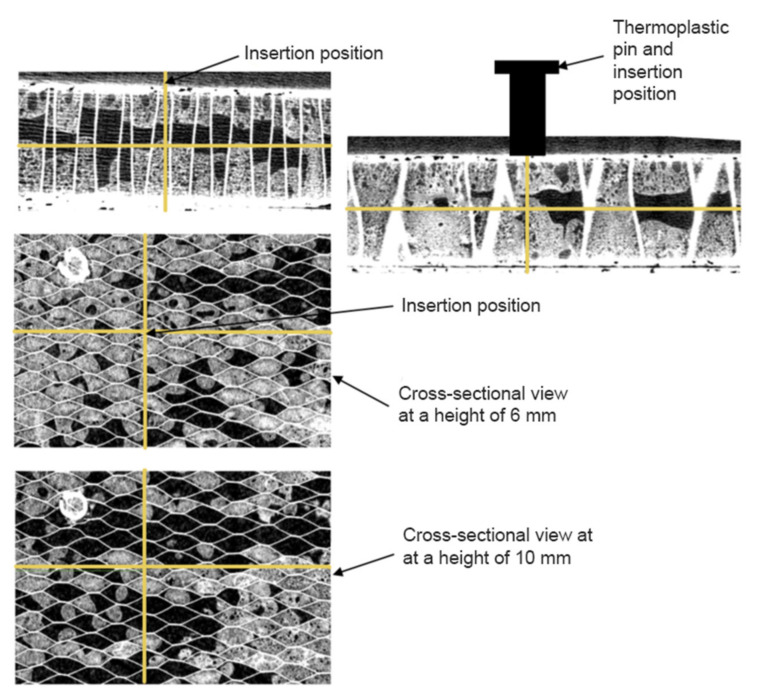
Computer tomography (CT) scanned sandwich structure with corresponding insertion position highlighted with yellow coordinate lines.

CT scans were analysed before and after the insertion process at the same position for validation. Several cross-sections of the sandwich structure were imaged to replicate an approximated structure in the impact model. Figure 5 presents a detailed view of the sandwich structure. It was possible to examine the amount of foam, the porosity of the foam, and its position on the cell structure. The insertion position in the substrate is highlighted with yellow coordinate lines to create a three-dimensional (3D) impression from different views (front view, top view, and side view). The geometry of the sandwich structure was reconstructed in detail because of the high variability of the internal sandwich structure and its strong influence on the process outcome. For this purpose, CT scans of the substrate sample were cut into eight slices in the thickness direction, and the sandwich geometry was represented as a layered structure with seven levels. At every level, the honeycomb cell walls were presented in a pattern corresponding to the sandwich structure in the insertion position (Figure 5).

The wall thickness (0.5 mm) was measured on the basis of CT scans. The internal space in each honeycomb cell at every level was filled with a 3D hexagonal prism domain, depending on the foam volume content in this region. The internal space of the cell was filled if the CT scan analyses showed that foam occupied more than 50% of the 3D hexagonal prism volume. Performing simplification of the foam distribution to FEM geometry allows the consideration of relatively large void areas inside the honeycomb, neglecting small local variations. The top and bottom composite surfaces were presented as two-dimensional shell structures defined as the skin of the 3D sandwich domain. The proposed method, for the simplified representation of a real 3D lightweight structure, considers non-uniform foam distribution inside the honeycomb with the composite face sheets, and its block-wise domain allows sweeping of the triangular mesh on the face sheets through the thickness direction, both of which provide an increased calculation accuracy in the impact region and reduce computational time.

**Figure 6:**
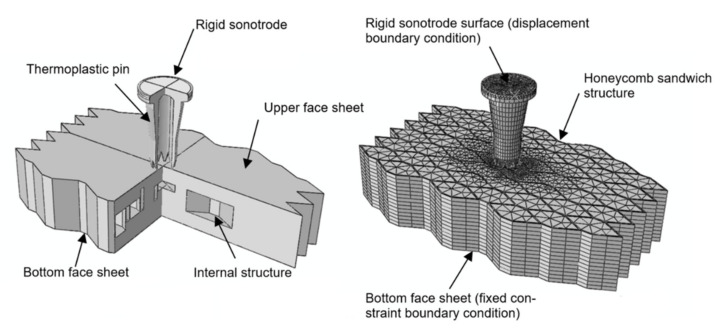
Overview of model assembly (thermoplastic pin, face sheet and internal structure) and resulting mesh with two node-based boundary conditions.

The model assembly consists of three parts: ultrasonic sonotrode, thermoplastic pin, and sandwich structure (Figure 6). The sonotrode material is titanium alloy (Ti-6Al-4V) and is therefore significantly stiffer than the pin. Accordingly, the sonotrode is considered to be a rigid surface with a shape corresponding to the sonotrode used in the experiment. The composite face sheets were defined as skins that are bonded to the surface of the three-dimensional sandwich domain via a tie constraint with consistent mesh. Skin reinforcements influence contact calculations through the change of contact penalty stiffness depending on the skin thickness. General contact in ABAQUS/Explicit was defined for all the external surfaces. General contact enforces contact constraints using a penalty contact method, which searches for node-into-face penetrations. For node-to-face contact, forces depend on the penetration distance. They were applied to the slave nodes to oppose the penetration, while equal and opposite forces act on the master surface at the penetration point.

The pins domain was meshed using 12′647 linear coupled temperature-displacement tetrahedron elements. The substrate’s domain was meshed using 62′656 linear coupled temperature-displacement triangular prism elements and composite shells were meshed with 3′194 triangular elements. Both sandwich and pin domains were meshed using sweep meshing routine, face sheets were meshed with free triangular mesh. The average volume of the pin’s elements is 0.95 mm^3^, of the substrate’s elements is 1.41 mm^3^ and the average area of the face sheet elements is 1.24 mm^2^. Abaqus/Explicit automatically evaluates the time step on the base of the minimal element size and mechanical properties of the considered domains. Therefore, proposed finite-element mesh is a result of the compromise between the simulation accuracy and total calculation time. Defined finite-element mesh provides an average time step being equal to 2 × 10^−8^ s, which leads to approximately 25 million iterations for the simulation of 0.5 s of the joining process. The numerical model was solved using 16 processors within 27 h. Decrease of the average element size leads to the significant increase of the calculation time, while increase of element size leads to no solver convergence due to the hour-glassing problem.

### 3.2. Boundary Condition Reconstruction

The displacement and its derivatives were set to zero and the temperature was set to 20 °C for all domains at the beginning of the simulation. The model contained two node-based boundary conditions: displacement for the rigid sonotrode and fixed constraint (zero displacements and rotations) for the sandwich bottom face sheet (see Figure 6). The vertical displacement of the rigid sonotrode was defined based on the experimental data. It takes into account the linear displacement of the sonotrode, oscillation frequency, and amplitude according to the expression

(9) $$u_{horn}=u_{web}+A_{web}sin\left(2\pi \omega t\right).$$

Here, $u_{web}$ is the vertical displacement of the sonotrode measured by the ultrasonic system, $A_{web}$ is the time-dependent value of the oscillation amplitude, and ω is the oscillation frequency. Displacements in other directions, as well as all the rotations were set to zero.

### 3.3. Stress and Temperature Distribution

**Figure 7:**
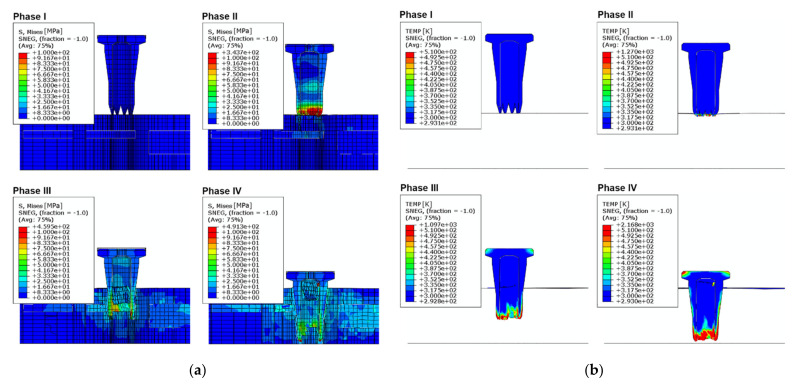
Simulation of the MM-Welding^®^ LiteWWeight^®^ process: (**a**) stress distribution within the four process phases; (**b**) Temperature distribution within the four process phases.

Figure 7 presents an overview of the resulting thermal-mechanical numerical model. The material properties are defined according to the experimental studies and are presented in Appendix A (Table A1). Figure 7a shows the stress distribution in the pin and substrate. The maximum stresses arise in the pin tips at the end of phase II (maximum stress time corresponds to the first reaction force peak time, when the collapse of the face sheet occurs, see Figure 4) and phase IV (time corresponds to the second reaction force peak, pin reaches the bottom of the substrate, see Figure 4).

The solution of the heat-transfer equations is shown in Figure 7b. Most of the pins plastic deformations and friction arise in the tip area owing to the permanent contact with the substrate during the pins’ movement, which leads to a temperature increase in that region. Heating of the head of the pin is explained by the friction between the head and sonotrode internal walls. The simulated temperature distribution corresponds to the melted areas observed after the process. A detailed comparison between the measured and simulated temperatures is provided later on.

### 3.4. Reaction Forces

**Figure 8:**
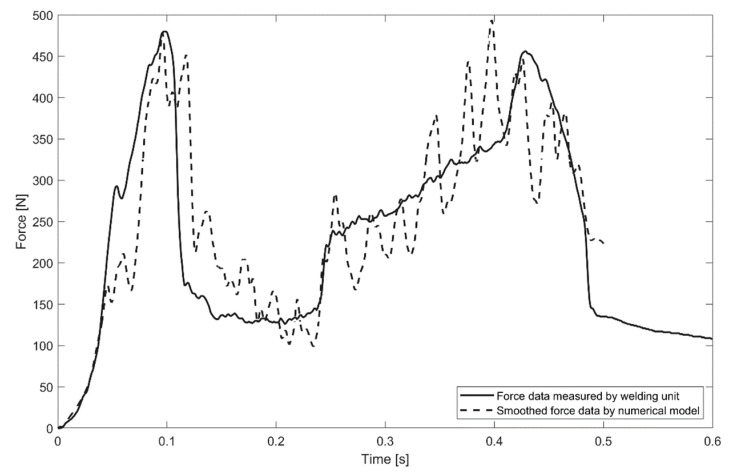
Numerically modelled and experimentally measured reaction force of the sonotrode.

The reaction force curve represents the main phases of the process (Figure 4). Therefore, the proposed numerical model should provide the same values of the reaction force as measured for the corresponding process phase. The sampling rate for the reaction force evaluation in the simulation was equal to 1/10 of the oscillation period. The reaction force signal provided by the ultrasonic welder was smoothed by a Gaussian filter, as described in Figure 3; for the comparison of simulated and measured data, the simulated reaction force curve was, therefore, smoothed by an identical filter. A comparison of the measured and simulated smoothed force values with time is presented in Figure 8. The calculated reaction force provided a reasonable correlation with the measured reaction force both qualitatively and quantitatively. The oscillations within the numerically modelled force signal arose from the element deletion criteria.

### 3.5. Damage Rate

A ‘partial insertion’ study was performed to measure the destruction rate of the pins tips on different phases of the process. The destruction rate in the final phase of the process plays a significant role in the formation of an interlock between the pin and bottom composite face sheet. A high destruction rate leads to the shortening of the pins body, and possibly to the situation where the pin does not reach the bottom face sheet and does not form an interlock, thereby significantly reducing the joint quality. On the other hand, a low destruction rate could lead to penetration of the bottom face sheet by the tips, which is unacceptable in terms of the visual appearance.

In the frame of the ‘partial insertion’ study, various collapse lengths were set in a series of trials. After partial insertion, the pin was pulled out and the reduction in the tip height was measured. Owing to the strong influence of the internal structure on the process, the pin lost different percentages of the original tip’s height with the same collapse distance at different insertion locations. However, in most cases, the pin destruction rate increased significantly after the upper face sheet collapsed and after reaching the bottom face sheet. In general, the pin-tip damage rate evolution can be described in four steps:

- Before the upper face sheet collapse (collapse length is 0–1.5 mm): tips were partially melted and slightly deformed; approximate loss of original tip’s height was 10%.
- After the upper face sheet collapsed (collapse length is 1.5–3 mm): tips were partially destroyed; approximate loss of original tip’s height was 10–30% depending on local stiffness under a single tip.
- Before the pins reached the bottom face sheet (collapse length is 3–16 mm), the destruction rate was slightly increased. Since the substrate (foam) material was much softer than the pins, the pin did not meet significant resistance during its insertion through the core of the sandwich structure. However, during this step, the temperature of the tips increased significantly owing to friction.
- After the pins reached the bottom face sheet (collapse length is 18 mm): the tips lost most of their original height.

**Figure 9:**
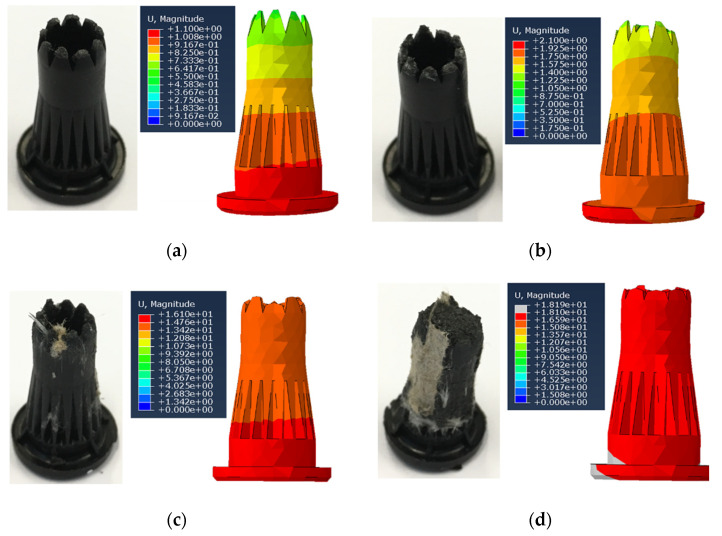
Observed and simulated pin damage rate at various collapse lengths: (**a**) Collapse length of 1 mm (before face sheet collapse); (**b**) Collapse length of 2 mm (after face sheet collapse); (**c**) Collapse length of 16 mm; (**d**) Maximum collapse length of 18 mm.

A comparison between the results of the ‘partial insertion’ study and simulation for the key steps of the joining process is presented in Figure 9. The correlation of the numerical model and experimental trials, by verifying the damage behaviour during the insertion process, showed a reliable quantitative result. The simulation of the tip destruction during the joining process allowed the optimisation of the pin design to provide the maximum interlocking surface without penetration of the bottom face sheet. The optimisation of the pins design based on the numerical models is the subject of future studies.

### 3.6. Hammering Coefficient

The idea of calculating the hammering coefficient is based on the determination of the contact and non-contact points within the measured signals. With the labelled points, the proportion can be calculated and referenced as the hammering coefficient. First, both data arrays (sonotrode and pin) were normalised by polynomial regression. Subsequently, the signal for clean oscillations was evaluated. The next step was to sum up the distances between the pin and the sonotrode and divide it by an averaged value. Then, the ‘contact’ and ‘non-contact’ points were defined and the occurrence was calculated. The hammering coefficient was evaluated by dividing both the sums. To date, this approach is the most robust and repeatable method.

**Figure 10:**
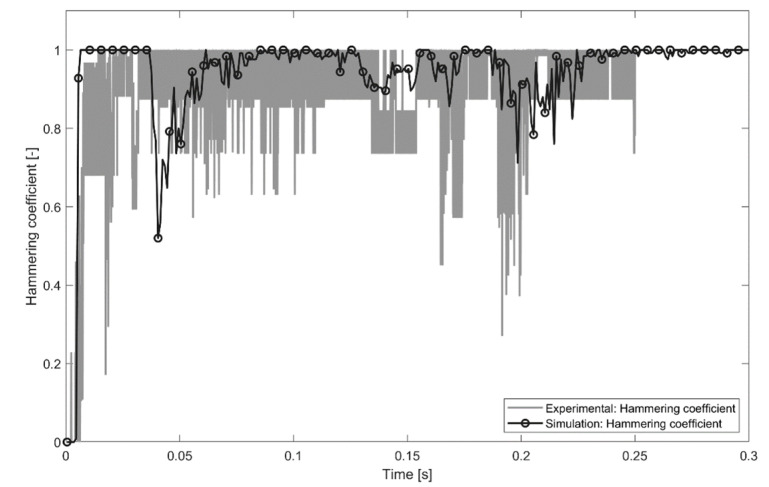
Hammering coefficient calculated on base of MATLAB point tracker and FEM.

Evaluation of the hammering coefficient is an important step in understanding sonotrode-pin interactions during the process. For the experiment, the hammering coefficient was evaluated using the MATLAB tracking algorithm, as described in the experimental methods. The hammering coefficient was also evaluated on the basis of the simulated reaction force. When the reaction force is equal to 0, there is no contact between the pin and sonotrode. The hammering coefficient value within the current time range can be evaluated as the ratio of non-zero force values to the total amount of force values. A comparison between the measured and simulated hammering coefficients is shown in Figure 10. The MATLAB tracking algorithm is not able to measure the hammering coefficient for the whole process, since no oscillations were existing before 0.05 s, and most of the tracking points were covered by the substrate and were not observable with the high-speed camera after 0.25 s.

Both FEM and MATLAB tracking algorithms show that after the ultrasonic vibrations started, the hammering coefficient was close to 1 (full contact) with small local reductions. The local hammering coefficient predicted by the FEM and MATLAB tracking algorithms did not always correlate with each other. This could be explained by the limited accuracy of the implemented methods (coarse finite-element mesh or low resolution of the high-speed camera) or by the inaccuracy of the substrate geometry reconstruction since local variation in the substrate’s foam content plays a significant role in the pins’ movement.

### 3.7. Temperature Distribution

**Figure 11:**
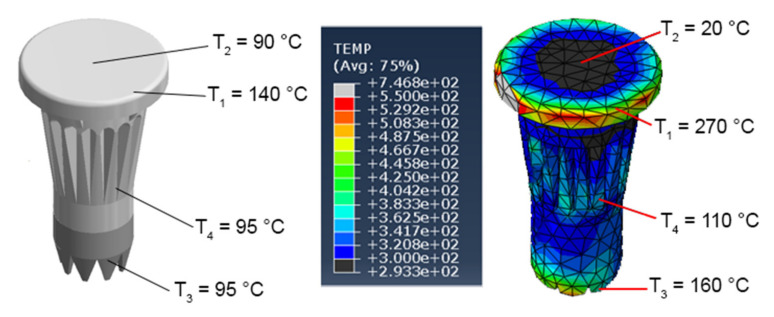
Temperature measurement after the insertion and pulling out and simulated temperature after the insertion.

Abaqus allows the consideration of heat from plastic deformation and friction. The Abaqus results for temperature are presented in Figure 11. In the current study, no direct temperature measurements were possible based on the fast process. Temperature measurements were performed immediately after insertion (the pin was pulled out shortly after the insertion and temperature was measured in several pin regions) for the comparison of the temperature distribution in the experiment and simulation. The difference between the experiment and simulation could be explained by the time elapsed from the insertion end to the measurement. During this time, temperature redistribution within the pin domain occurred and free convection with the room environment cooled the pin. However, spatial distribution of the temperature in the simulation was close to that in the experiment, and the pins head in experiment had signs of melting, which meant that the temperature there was higher than 240 °C during the joining.

The temperature increase due to the elastic deformations was calculated using Equation (5) for the heat from the ultrasonic energy dissipation. According to this equation, the temperature grew only by about 0.1 degree after 0.5 s, which was significantly lower than the heat from the plastic deformations and friction and was, therefore, neglected in further calculations of the degree of interlock.

### 3.8. Connection Quality Evaluation

**Figure 12:**
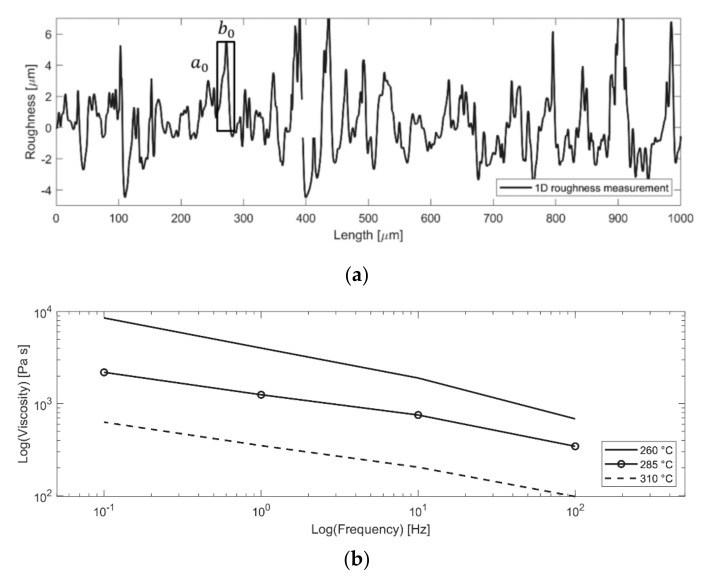
(**a**) One−dimensional surface roughness measurement on the ‘connecting’ surface of the pin; (**b**) Frequency- and temperature-dependent viscosity measurement of the pins material PA66 GF30.

Figure 12a shows a one-dimensional representation of the surface roughness of the pins, resulting in $R_{z}=6.44\;\mu m$ and $R_{a}=0.93\;\mu m$. The measurements were used to calculate the degree of interlock. Therefore, the approximated proportion $a_{0}/b_{0}=0.3$ was extracted for use in Equation (8). Furthermore, Figure 12b presents the frequency- and temperature-dependent viscosity measurements of the PA66 GF30 pin material.

**Figure 13:**
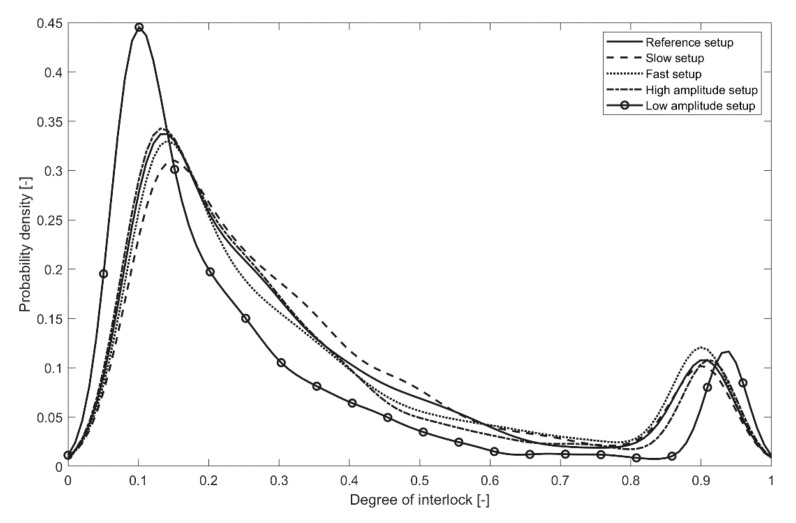
Degree of interlock distribution among nodes for the performed setups.

The sonotrode boundary conditions were reconstructed for every setup on the basis of the exported data according to Equation (9). Since Abaqus software does not support the calculation of the connection quality, a MATLAB routine was developed for the evaluation of the degree of interlock on the base of thermal and stress distribution exported from the finite-element model. Thus, the Abaqus solution for the temperature and pressure was exported for every finite element (except the head region and elements that were destroyed during the process) for every time step starting from the Phase III (after pins vertical movement slows down), and implemented into Equation (8) for the degree of interlock. According to the simulated and experimentally-observed temperature distributions, only the tip and energy director area of the pin were subjected to melting. Therefore, only a small percentage of all nodes reached a fully interlocked state ($D_{ic}=1$). The degree of interlock distribution among the defined nodes is presented in Figure 13 with a smoothed probability density function.

The calculated average degrees of the interlock and measured pullout forces are presented in Table 2. Experimental trials show that the process did not depend significantly on the small variations in the parameter setup. Only the ‘Low Amplitude’ configuration demonstrated significant (10%) reduction of the average pullout force, while other setups demonstrated small (1–3%) variation of the pullout force in comparison with the ‘Reference’ setup. The developed numerical model predicted 16% reduction (vs. 10% for the experimental trials) of the average pullout force for the ‘Low Amplitude’ setup, which was considered a reasonable agreement with the experiment. Nevertheless, the numerical model was not able to predict a small (3%) reduction in the average pullout force for the ‘Fast’ setup. It also predicted a small (4%) reduction in the average pullout force for the ‘High Amplitude’ setup, while the experiments showed a small increase (1%). The developed numerical model predicted only a significant change in the average pullout force depending on the process setup. Therefore, the proposed numerical approach can be used to define the process setup in the case of new pin or substrate designs, where significantly different setups must be tested, although the model was not able to track the changes in the final joint quality within the small variation of the parameter setup. Thus, a prediction accuracy between 94–99% depending on the setup was achieved.

## 4. Conclusions

In this study, the MM-Welding^®^ LiteWWeight^®^ technology was presented, and a thermo-mechanical numerical model that predicts the joining quality was developed. Based on the results obtained, the following conclusions can be highlighted:

- Material properties of the pin and sandwich were defined using various experimental measuring techniques.
- The geometry in the numerical model was reconstructed based on computed tomography scans to ensure identical process conditions, pin position, and local foam distribution.
- The numerical model successfully calculated the damage rates and heat from friction and plastic deformations of the pin and sandwich structures.
- The model was verified and validated based on comparative experiments.
- The routine for the prediction of the connection quality from the finite-element model temperature and stress distribution was derived based on the degree of the interlock approach, which only showed reasonable agreement for significantly different process setups resulting in a prediction accuracy between 94–99%. Despite this fact, the methodology applied suits the potential for lowering the effort towards new pin and sonotrode materials.
- The numerical simulation and data analyses conducted during the process enabled the discrimination and identification of critical process and design features, such as hammering, damage rate or reaction force.

Presented results will not only accelerate process development cycles, but also provide the potential for an advanced online process quality monitoring system. Predictive capabilities of the developed model enable the possibility of numerical-based processes and pin geometry optimization, which is the focus of the future studies.

## Figures and Tables

**Table 1:** Ultrasonic process setup for assessment of the connection quality.

Setup	Insertion Speed [mm/s]	Amplitude [μm]
Reference	45	76
Slow	40	76
Fast	50	76
High Amplitude	45	92
Low Amplitude	45	62

**Table 2:** Measured pullout forces and corresponding simulated average degree of interlock (DIC).

Setup	Average Pullout Force (% to Reference Value)	Average Degree of Interlock (% to Reference Value)
Reference	530.2 N (100%)	0.3762 (100%)
Slow	525.83 N (99%)	0.3824 (101%)
Fast	511.76 N (97%)	0.3790 (101%)
High Amplitude	534.43 N (101%)	0.3600 (96%)
Low Amplitude	477.8 N (90%)	0.3166 (84%)

## Data Availability

The data presented in this study is available on request from the corresponding author.

## Supplementary Materials

The following are available online at https://www.mdpi.com/article/10.3390/ma14206005/s1, Equations (S1)–(S24): Governing equations.

## Appendix A

**Table A1:** FEM material definitions and contact properties.

Domain/Surface	Pin	Face Sheet	Honeycomb Cell	Foam
Element type	A 4-node thermally coupled tetrahedron, linear displacement and temperature	A 3-node thermally coupled triangular thin shell, finite membrane strain.	A 6-node thermally coupled triangular prism, linear displacement, and temperature.	
Density [tonne/mm^3^]	1.34 × 10^−9^	1.7 × 10^−9^	1.3 × 10^−9^	1.15 × 10^−9^
Elastic modulus E [MPa]	Elastic modulus [MPa]/Temperature [K] 5500/303; 4051/323; 2357/348; 2085/373; 1784/398; 1587/423; 1314/473; 487/533	4550	400	300
Poisson’s ratio ν [1]	0.35	0.35	0.33	0.2
Plastic properties	Stress σ [MPa]/Strain ε [1] 5/0; 10/0.005; 20/0.007; 30/0.01; 40/0.015; 50/0.017; 60/0.02; 70/0.025; 80/0.027; 90/0.03; 100/0.04; 110/0.045; 120/0.06; 130/0.07; 150/0.1	-	Stress σ[MPa]/Strain ε [1] 30/0 55/0.39	Stress σ[MPa]/Strain ε [1] 25/0 30/0.39
Damage criteria	Ductile: displacement criteria	Hashin	Ductile: displacement criteria	Ductile: displacement criteria
Damage initiation	Equivalent plastic strain: Initiation strain [1]/Temperature [K] 0.5/300 0.4/400 0.3/500	Tensile strength in the fibre direction $X^{T}$/Compressive strength in the fibre direction $X^{C}$/Tensile strength in the transverse direction $Y^{T}$/Compressive strength in the transverse direction $Y^{C}$/Longitudinal shear strength $S^{L}$/Transverse shear strength $S^{T}$ 138/80/138/80/100/100	Initiation strain [1]: 0.5	Initiation strain [1]: 0.5
Damage evolution	Effective plastic displacement: 0.4	Energy: 1 × 10^−9^	Effective plastic displacement: 0.001	Effective plastic displacement: 0.001
Thermal conductivity [W/mm/K]	Conductivity [W/mm/K]/Temperature [K] 0.36/300 0.5/400 0.6/500	0.1	0.1	0.1
Specific heat [J/K/tonne]	1.5 × 10^9^	1 × 10^9^	1 × 10^9^	1 × 10^9^
Inelastic heat fraction $\eta_{pl}$ [1]	0.75	-	0.75	0.75
Friction coefficient $\mu_{fr}$ [1]	0.5			
Fraction of frictional work $\eta_{fr}$ [1]	1			
Contact stiffness [MPa]	850			
