# Supplementary material for: Experimental and Numerical Development on Multi-Material Joining Technology for Sandwich-Structured Composite Materials
Source: Materials (Basel). 2021 Oct 12;14(20):6005. doi: 10.3390/ma14206005 (PMC8537860; doi:10.3390/ma14206005)
Supplement: Supplementary file 1 [file materials-14-06005-s001.zip › materials-1380174-supplementary.pdf]

Supplementary Materials

Governing Equations

The following section supplements 2.4 Numerical methods. A ductile damage material model was implemented for the pin, honeycomb cells, and foam. For every material, the equivalent plastic strain at the damage initiation was defined (Appendix A). Once the damage initiation criterion was reached, the effective plastic displacement $\bar{u}^{pl}$ needed to be specified, and its evolution is defined by

$$\dot{\bar{u}}^{pl} = L\dot{\varepsilon}^{pl}, \tag{S1}$$

where L is the characteristic length of the element. The evolution of the damage variable with the relative plastic displacement is specified in a linear form:

$$\dot{d} = \dot{\bar{u}}^{pl}/\bar{u}^{pl}, \tag{S2}$$

where d is the damage variable, which provides the degradation of the stiffness according to

$$\sigma = (1 - d)\bar{\sigma} \tag{S3}$$

where $\bar{\sigma}$ is the effective stress tensor. The material loses its load-carrying capacity when $d = 1$.

The Hashin damage model predicted anisotropic damage in fibre-reinforced composite face sheets and considered four different failure modes: fibre tension, fibre compression, matrix tension, and matrix compression.

Fibre tension ($\hat{\sigma}_{11} \geq 0$):

$$\left(\frac{\hat{\sigma}_{11}}{X^T}\right)^2 + \left(\frac{\hat{\tau}_{12}}{S^L}\right)^2 = \begin{cases} \geq 1 : \text{failure} \\ > 1 : \text{no failure} \end{cases}, \tag{S4}$$

Fibre compression ($\hat{\sigma}_{11} < 0$):

$$\left(\frac{\hat{\sigma}_{11}}{X^C}\right)^2 = \begin{cases} \geq 1 : \text{failure} \\ < 1 : \text{no failure} \end{cases}, \tag{S5}$$

Matrix tension ($\hat{\sigma}_{22} \geq 0$):

$$\left(\frac{\hat{\sigma}_{22}}{Y^T}\right)^2 + \left(\frac{\hat{\tau}_{12}}{S^L}\right)^2 = \begin{cases} \geq 1 : \text{failure} \\ < 1 : \text{no failure} \end{cases}, \tag{S6}$$

Matrix compression ($\hat{\sigma}_{22} < 0$):

$$\left(\frac{\hat{\sigma}_{22}}{2S^T}\right)^2 + \left[\left(\frac{Y^C}{2S^T}\right)^2 - 1\right]\frac{\hat{\sigma}_{22}}{Y^C} + \left(\frac{\hat{\tau}_{12}}{S^L}\right)^2 = \begin{cases} \geq 1 : \text{failure} \\ < 1 : \text{no failure} \end{cases}. \tag{S7}$$

where X^T is the tensile strength in the fibre direction, X^C is the compressive strength in the fibre direction, Y^T is the tensile strength in the transverse direction, Y^C is the compressive strength in the transverse direction, S^L is the longitudinal shear strength, and S^T is the transverse shear strength. The values of these model parameters were defined from the experiments described in the first chapter and presented in Appendix A. $\hat{\sigma}$ is the effective stress tensor, which is calculated as follows:

$$\hat{\sigma} = M\sigma, \tag{S8}$$

where M is damage operator defined as

$$M = \begin{bmatrix} 1/(1-d_f) & 0 & 0 \\ 0 & 1/(1-d_m) & 0 \\ 0 & 0 & 1/(1-d_s) \end{bmatrix}, \tag{S9}$$

where d_f, d_m, and d_s are internal damage variables that characterise fibre, matrix, and shear damage, respectively.

$$d_f = \begin{cases} d_f^t, & \hat{\sigma}_{11} \geq 0 \\ d_f^c, & \hat{\sigma}_{11} < 0 \end{cases}, \tag{S10}$$

$$d_m = \begin{cases} d_m^t, & \hat{\sigma}_{22} \geq 0 \\ d_m^c, & \hat{\sigma}_{22} < 0 \end{cases}, \tag{S11}$$

$$d_s = 1 - (1-d_f^t)(1-d_f^c)(1-d_m^t)(1-d_m^c). \tag{S12}$$

Here, d_f^t, d_f^c, d_m^t, and d_m^c are the damage variables. When the damage criteria were met at an integration point, all the stress components were set to zero so that the material points were deleted. When all the material points at any one section of an element failed, the element was removed.

In Abaqus a nonlinear coupled system is solved using Newton's method [22]. An exact implementation of Newton's method involves a non-symmetric Jacobian matrix as follows

$$\begin{Bmatrix} K_{uu} & K_{u\theta} \\ K_{\theta u} & K_{uu} \end{Bmatrix} \begin{Bmatrix} \Delta u \\ \Delta \theta \end{Bmatrix} = \begin{Bmatrix} R_u \\ R_\theta \end{Bmatrix}, \tag{S13}$$

where Δu and Δu are the respective corrections to the incremental displacement and temperature, K_{uu}, $K_{u\theta}$, $K_{\theta u}$ and K_{uu} are submatrices of the fully coupled Jacobian matrix, and R_u and R_θ are the mechanical and thermal residual vectors, respectively.

Heat transfer equations are integrated using the explicit forward-difference time integration rule

$$\theta_{i+1}^N = \theta_i^N + \Delta t_{i+1} \dot{\theta}_i^N, \tag{S14}$$

where θ^N is the temperature at node N and subscript i refers to the increment number. The current temperatures are obtained using known values of $\dot{\theta}_i^N$, which are calculated in the beginning of time increment

$$\dot{\theta}_i^N = (C^{NJ})^{-1} \left(Q_i^J - F_i^J \right), \tag{S15}$$

in which C^{NJ} is the lumped capacitance matrix, Q_i^J is the applied nodal source vector, and F_i^J is the internal flux vector. The mechanical solution response is obtained using the explicit central-difference integration rule with a lumped mass matrix

$$\dot{u}_{i+0.5}^N = \dot{u}_{i-0.5}^N + 0.5(\Delta t_{i+1} + \Delta t_i) \ddot{u}_i^N, \tag{S16}$$

$$u_{i+1}^N = u_i^N + \Delta t_{i+1} \dot{u}_{i+0.5}^N. \tag{S17}$$

Here u^N is degree of freedom. Acceleration at the beginning of the increment are computed as

$$\ddot{u}_i^N = (M^{NJ})^{-1} \left(P_i^J - I_i^J \right), \tag{S18}$$

where M^{NJ} is the mass matrix, P^J is the applied load vector, and I^J is the internal force vector. Since both the forward-difference and central-difference integrations are explicit, the heat transfer and mechanical solutions are obtained simultaneously by an explicit coupling. The stability limit for both central-difference and forward-difference operators is obtained by choosing

$$\Delta t \leq min(2/\omega_{max}, 2/\lambda_{max}), \tag{S19}$$

in which $\omega_{max}$is the highest frequency in the system of equations of the mechanical solution response and λ_{max} is the largest eigenvalue in the system of equations of the thermal solution response. An approximation to the stability limit for the forward-difference operator in the thermal solution response is given by

$$\Delta t \approx L_{min}^2/2\alpha, \tag{S20}$$

where L_{min} is the smallest element dimension in the mesh and α is the thermal diffusivity of the material, which depends on the material's thermal conductivity, density, and specific heat. Stability limit of the time increment for the equations of body motion is

$$\Delta t \approx L_{min}/c_d, \tag{S21}$$

in which c_d is the dilatational wave speed.

The total stress is defined from the total elastic strain as

$$\sigma = D^{el}(\varepsilon^{el} + \varepsilon^{pl}), \tag{S22}$$

where D^{el} is the fourth-order elasticity tensor, and ε^{el} is the total elastic strain. Elastic properties of pin, honeycomb cells and foam are considered as isotropic. The stress-strain relationship is given by

$$\begin{Bmatrix} \varepsilon_{11} \\ \varepsilon_{22} \\ \varepsilon_{33} \\ \gamma_{12} \\ \gamma_{13} \\ \gamma_{23} \end{Bmatrix} = \begin{bmatrix} 1/E & -v/E & -v/E & 0 & 0 & 0 \\ -v/E & 1/E & -v/E & 0 & 0 & 0 \\ -v/E & -v/E & 1/E & 0 & 0 & 0 \\ 0 & 0 & 0 & 1/G & 0 & 0 \\ 0 & 0 & 0 & 0 & 1/G & 0 \\ 0 & 0 & 0 & 0 & 0 & 1/G \end{bmatrix} \begin{Bmatrix} \sigma_{11} \\ \sigma_{22} \\ \sigma_{33} \\ \sigma_{12} \\ \sigma_{13} \\ \sigma_{23} \end{Bmatrix}. \tag{S23}$$

The elastic properties are defined by the Young's modulus E, and the Poisson's ratio v. The shear modulus, G, is defined as $G = E/2(v + 1)$. These parameters defined as constants, except the Young`s modulus of pin, which is given as functions of temperature as presented in Appendix A. For the shell elements of composite face sheets an orthotropic material is defined with the following stress-strain relations

$$\begin{Bmatrix} \varepsilon_1 \\ \varepsilon_2 \\ \gamma_{12} \end{Bmatrix} = \begin{bmatrix} 1/E_1 & -v_{12}/E_1 & 0 \\ -v_{12}/E_1 & 1/E_2 & 0 \\ 0 & 0 & 1/G_{12} \end{bmatrix} \begin{Bmatrix} \sigma_{11} \\ \sigma_{22} \\ \tau_{12} \end{Bmatrix}. \tag{S24}$$

General contact was defined in Abaqus/Explicit for the simulation of contact and interaction problem. All surfaces are defined automatically. This default surface contains all exterior element faces, all analytical rigid surfaces and all edges in the model, as well as the nodes attached to these faces and edges. The general contact algorithm activates and deactivates contact faces and contact edges in the contact domain based on the failure status of the underlying elements.
